# 206. Prevalence of liver abscess in patients with *Klebsiella pneumoniae* bacteremia during a 5-year experience in a tertiary hospital in Thailand

**DOI:** 10.1093/ofid/ofad500.279

**Published:** 2023-11-27

**Authors:** Natthapol Kiatkangwanchon, Chris Fujitnirun

**Affiliations:** Chulalongkorn University, Bangkok, Krung Thep, Thailand; Bhumibol Adulyadej Hospital, Bangkok, Krung Thep, Thailand

## Abstract

**Background:**

The incidence of liver abscess in Thailand has increased in number nowadays.

*K. pneumoniae* is the most common cause of pyogenic liver abscess in the recent large series. Liver abscess can be a complication of *K. pneumoniae* bacteremic patients. We aimed to study the prevalence of liver abscess in patients with *K. pneumoniae* bacteremia and the comparison in clinical and laboratory characteristics of patients with *K. pneumoniae* between a group with liver abscess and the other without liver abscess.

**Methods:**

This is a retrospective study; data were collected in Bhumibol Adulyadej Hospital among patients who were diagnosed with *K. pneumoniae* bacteremia between January 2014 – December 2019 and compared the difference in clinical and laboratory characteristics of patients between a group with liver abscess and the other without liver abscess.

**Results:**

Of All 758 patients with *K. pneumoniae* bacteremia, 335 (44.19%) patients were done abdominal imaging, and 37 (4.9%) patients were found with liver abscess. The mean age was 65 years old, and most were male. Comorbidities were present in almost half of the patients including diabetes (118 patients, 35.22%), previous hepatobiliary surgery (21, 6.26%), and history of cancer (70, 20.90%). The other demographic data are shown in Table 1. Patients with liver abscess had diabetes (54.1% vs 29.3%, p-value = 0.082) and fever (91.89% vs 76.84%, p-value = 0.056) more than patients without abscess, however only 29.7% of patients with liver abscess had abdominal pain. Elevated transaminases above 2 times the upper limit of normal range are more frequent among patients with liver abscess than patients without abscess, for AST; 54.1%vs 35.30% (p-value = 0.026) and ALT; 40.5% vs 25.20% (p-value = 0.048). Clinical and laboratory characteristics are shown in Table 2.

**Table 1:**
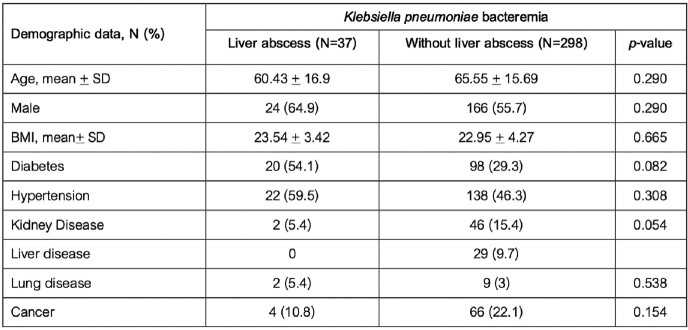
Demographic data of patients with K. pneumoniae bacteremia

**Table 2:**
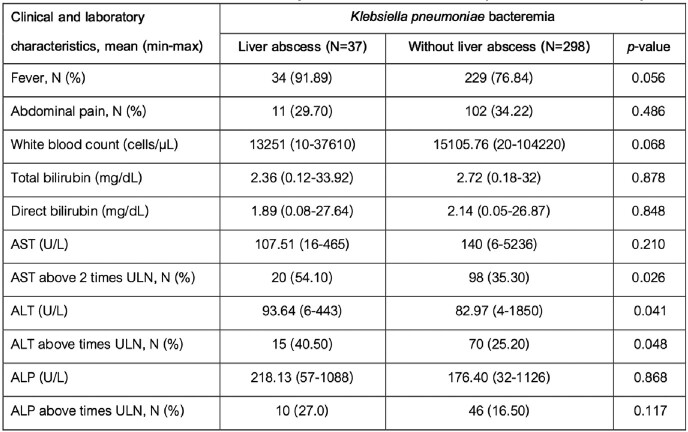
Clinical and laboratory characteristics of patients with K. pneumoniae bacteremia

**Conclusion:**

The prevalence of liver abscess in patients with *K. pneumoniae* bacteremia is 37 from 758 (4.9%). Liver abscess should be suspected in *K. pneumoniae* bacteremic patients who have elevated transaminases above 2 times the upper limit of normal range regardless of abdominal pain.

**Disclosures:**

**All Authors**: No reported disclosures

